# Comparison of Structure and Local Dynamics of Two Peptide Dendrimers with the Same Backbone but with Different Side Groups in Their Spacers

**DOI:** 10.3390/polym12081657

**Published:** 2020-07-25

**Authors:** Sofia E. Mikhtaniuk, Valeriy V. Bezrodnyi, Oleg V. Shavykin, Igor M. Neelov, Nadezhda N. Sheveleva, Anastasia V. Penkova, Denis A. Markelov

**Affiliations:** 1 St. Petersburg National Research University of Information Technologies, Mechanics and Optics (ITMO University), Kronverkskiy pr. 49, 197101 St. Petersburg, Russia; mikhtanyuk@mail.ru (S.E.M.); v.v.bezrodniy@mail.ru (V.V.B.); kupala-89@mail.ru (O.V.S.); i.neelov@mail.ru (I.M.N.); 2 St. Petersburg State University, 7/9 Universitetskaya nab., 199034 St. Petersburg, Russia; shevelevann@gmail.com (N.N.S.); a.penkova@spbu.ru (A.V.P.)

**Keywords:** peptide dendrimer, computer simulation, molecular dynamics, NMR, spin-lattice relaxation time, charged macroion, zeta potential

## Abstract

In this paper, we perform computer simulation of two lysine-based dendrimers with Lys-2Lys and Lys-2Gly repeating units. These dendrimers were recently studied experimentally by NMR (Sci. Reports, 2018, 8, 8916) and tested as carriers for gene delivery (Bioorg. Chem., 2020, 95, 103504). Simulation was performed by molecular dynamics method in a wide range of temperatures. We have shown that the Lys-2Lys dendrimer has a larger size but smaller fluctuations as well as lower internal density in comparison with the Lys-2Gly dendrimer. The Lys-2Lys dendrimer has larger charge but counterions form more ion pairs with its NH${}_{3}^{+}$ groups and reduce the bare charge and zeta potential of the first dendrimer more strongly. It was demonstrated that these differences between dendrimers are due to the lower flexibility and the larger charge (+2) of each 2Lys spacers in comparison with 2Gly ones. The terminal CH${}_{2}$ groups in both dendrimers move faster than the inner CH${}_{2}$ groups. The calculated temperature dependencies of the spin-lattice relaxation times of these groups for both dendrimers are in a good agreement with the experimental results obtained by NMR.

## 1. Introduction

Dendrimers are spherical, regularly branched macromolecules [1,2]. They have a central core, branched repeating units, and a large number of terminal groups which are available for functionalization. Dendrimers (for example, polyamidoamine (PAMAM) and polyethylenimine (PEI) dendrimers) are widely used in many biomedical applications, for example, as antibacterial and antiviral agents [3] as well as nanocontainers for delivery of drugs [4,5], genes [6,7,8,9], and other nanoparticles [10].

At the same time, there are poly-L-Lysine (PLL) dendrimers that consist of natural lysine amino acid residues [11,12,13]. Due to the presence of these residues, such dendrimers have lower toxicity than PAMAM dendrimers. Therefore, PLL dendrimers are more suitable as antibacterial and antiviral agents [14], and as drug [15,16,17,18] and gene [19,20] delivery vehicles [21]. They have antiangiogenic properties that help to inhibit tumor growth [22]. Biodistribution of PLL dendrimers was studied in several papers [23,24]. Despite numerous applications in biomedicine of PLL dendrimers, their physico-chemical properties have been studied in a few works in comparison with commercially available PAMAM dendrimers. In particular, the systematic studies of a hydrodynamic radius and a radius of gyration ($R_{g}$) of PLL dendrimers of different generations (G=1–10) were performed only in dimethylformamide solvent [25,26]. The sizes and the local mobility of PLL dendrimers (G=2,4) in water at different temperatures have been recently studied by hydrodynamic and NMR methods [27,28,29]. Molecular dynamics (MD) simulation of PLL dendrimers of G=4 with modified terminal groups in water was also presented [30]. Systematic MD simulation of PLL dendrimers with unmodified terminal groups in water for G=1–5 at room temperature [31] and for G=2,4 at different temperatures [27,28,29] have been performed. Brownian dynamics (BD) simulation [32,33,34] and self-consistent field simulation [35] for study of these dendrimers were also applied.

Generally, peptide dendrimers [36,37,38,39,40,41] can consist of any combination of amino acid residues. Some authors include in this class any dendrimers (for example, PAMAM, PEI, etc.) with modified terminal groups containing amino acid residues [40,41]. At the same time, the most common peptide dendrimers still only have branched lysine amino acid residues in each repeating unit [36,37,38] while other amino acid residues or short linear peptides are chemically attached to terminal groups of a dendrimer [36,37]. The other possibility of creating new peptide dendrimers is an insertion of amino acids residues or short peptides as spacers between neighboring branching points inside dendrimers [38]. Application of peptide dendrimers in catalysis was described in [42,43,44], as antimicrobial peptides in [45,46,47], and for transdermal delivery in [48]. Applications of dendrimers for drug delivery were described in [49] and for gene delivery in [50,51,52,53,54,55,56]. Our study of siRNA delivery to normal and cancer cells using dendrimers simulated in this paper was described in [57], and using dendrimers of other amino acid composition but with the same distribution of charges along backbone of dendrimer in [58]. Computer simulation of peptide dendrimers was performed earlier by Brownian dynamics [59], molecular dynamics [60,61] methods, and by a numerical self-consistent field approach [62]. Synthesis and NMR study of some new peptide dendrimers with internally inserted amino acid spacers between each pair of branching points were also described in recent papers [63,64,65,66].

The main goal of the present paper is to investigate the structure and local orientational mobility of two peptide dendrimers having the same backbone but different volume and charge of side groups by MD method, and to compare their structural properties and orientational dynamics with the experimental results obtained earlier [63] using NMR spectroscopy. It is important to note that these dendrimers were tested as carriers for gene delivery [57].

## 2. Materials and Methods

**Figure 1:**
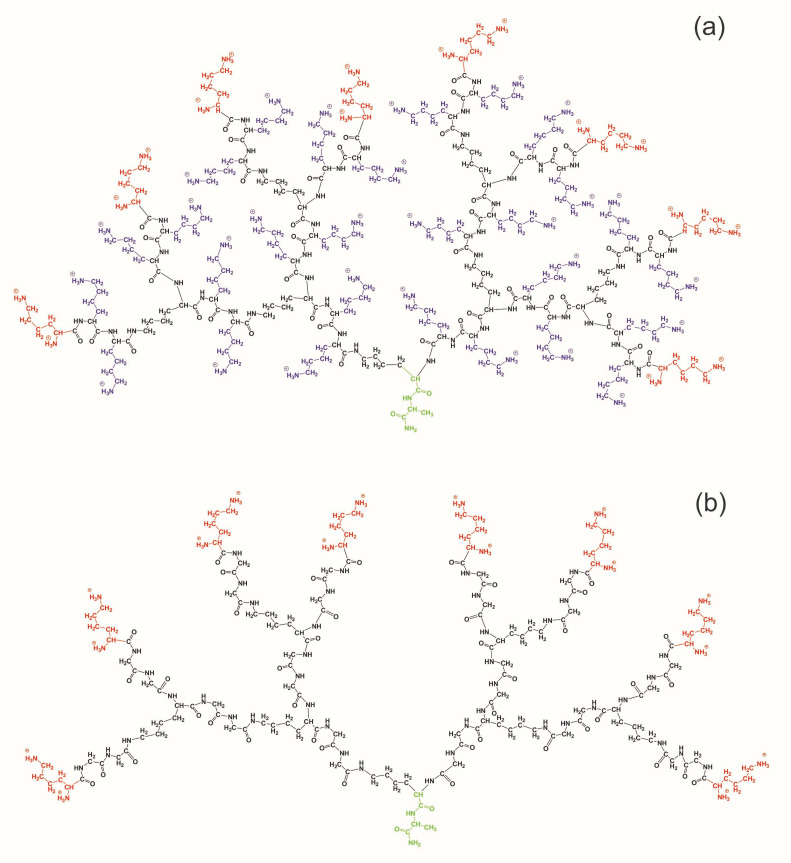
Chemical structure of Lys-2Lys (**a**) and Lys-2Gly (**b**) dendrimers. Cores of both dendrimers are marked by green color, backbones by black color, and terminal lysines by red color. The difference between the two dendrimers is the side segments of the Lys-2Lys dendrimer marked by violet color.

Two peptide dendrimers of the second generation with the same backbone but with different repeating units: Lys-2Gly or Lys-2Lys, were studied by computer simulation. The full atomic model, molecular dynamics method, and Gromacs package [67] were used. The structure of both dendrimers is shown in Figure 1. Both dendrimers have a core (marked by green color in Figure 1), a backbone (black), and terminal groups (red). The difference between two dendrimers is the bulky charged side groups of inserted 2Lys spacers (marked by violet) in Lys-2Lys dendrimer. Simulations were performed for each dendrimer in water with the explicit Cl-counterions. The number of those counterions (see Table 1) was equal to the number of charged groups in the dendrimer.

The molecular weight *M* of the Lys-2Lys dendrimer is essentially greater than *M* of the Lys-2Gly dendrimer (see Table 1) due to the massive side groups (marked by violet color in Figure 1) of the inserted 2Lys spacers. The charge of the Lys-2Lys dendrimer ($Q_{bare}$ = +44) with the additional charge of 2Lys insertions ($Q_{ins}$ = +28, because each inserted lysine residue $N_{ins}$ = 28 has a charge of the side NH${}_{3}$ group equal to +1) was also essentially larger than the charge of Lys-2Gly dendrimer ($Q_{bare}$ = +16) with 2Gly insertions ($Q_{ins}$ = 0, because each of the glycine residues $N_{ins}$ = 28 has no charge). Both dendrimers have the same numbers of terminal lysine groups, and their charges are equal to $N_{end}$ = 16 and $Q_{end}$ = +16, respectively (see Table 1).

The AMBER-99SB-ildn force field [68] was used in simulation. The potential energy of the system includes the deformation energy of the covalent bonds, the valence and the dihedral angles (including the improper dihedrals that maintain a plane conformation of peptide groups), and the energy of the van der Waals and electrostatic interactions. We used several computer programs, which were elaborated in previous papers, on MD [69,70,71] and BD simulations of dendrimers and dendrigrafts [72,73], linear polymers [74,75,76,77], polysaccharides [78], and peptides [79], as well as linear polyelectrolytes [80,81,82,83,84,85,86], for numerical calculations the properties of peptide dendrimers.

Calculations for each system included the structure optimization in vacuum, the initial equilibration using short MD simulations, and the long productive MD simulation run. The initial equilibration of both systems consisted of three stages: (1) the energy minimization of the system (a dendrimer with counterions in water) using the gradient descent method with a maximum number of steps of 50,000; (2) three runs of initial molecular dynamics simulation in the NVT-ensemble, where integration steps of the runs were 0.02, 0.2, and 2 fs, each run included 50,000, 50,000 and 500,000 steps respectively; and (3) one run of initial molecular dynamics simulation for 10 ns in NPT-ensemble where integration step was 1 fs. The second stage of initial equilibration was carried out in a cubic periodic cell with a fixed size. At this stage the temperature T=310 K was maintained by the modified Berendsen thermostat [87] with a time constant of τ = 0.4 ps. Moreover, the cubic periodic cell was used for the third stage of the initial equilibration. The pressure of 1 atm was maintained by the Berendsen barostat with the time constant τ = 0.5 ps. As a result, the systems with solvated Lys-2Lys and Lys-2Gly dendrimers were prepared. The characteristics of both dendrimers are shown in Table 1.

The productive run was performed by the molecular dynamics method with an integration step of 1 fs. Simulation was performed in the NPT ensemble. Constant temperatures of 280, 290, 300, 310, 320, and 340 K were maintained by the Nose–Hoover thermostat [88] with the time constant τ = 0.4 ps. A constant pressure of 1 bar was maintained by the Parrinello–Rahman barostat [89] with τ = 0.5 ps and with compressibility of water 4.9 × 10${}^{-5}$, 4.7 × 10${}^{-5}$, 4.5 × 10${}^{-5}$, 4.4 × 10${}^{-5}$, 4.4 × 10${}^{-5}$, 4.4 × 10${}^{-5}$, and 4.4 × 10${}^{-5}$ bar${}^{-1}$ at temperature 280, 290, 300, 310, 320, and 340 K, respectively [90]. Each system was simulated for 250 ns and the all atomic coordinates were recorded after each 100 fs.

## 3. Results and Discussion

### 3.1. The Global Characteristics

**Figure 2:**
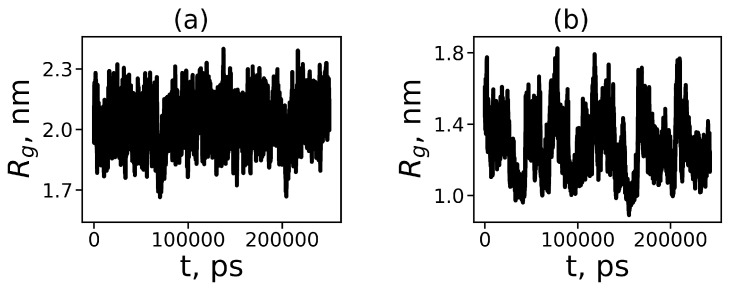
The time evolution of the mean-squared gyration radius $R_{g}\left(t\right)$ for (**a**) Lys-2Lys and (**b**) Lys-2Gly dendrimers at temperature T=310 K.

The characteristic size of a dendrimer can be estimated as a mean-squared gyration radius,

(1) $$R_{g}\;=\;{\left(\frac{1}{M}\sum_{i}m_{i}r_{i}^{2}\right)}^{1/2},$$

where $M,\;m_{i}$ are the molecular masses of the dendrimers and its *i*-th atom correspondingly and $r_{i}$ is the distance from the *i*-th atom to the center of mass of the dendrimer. The dependence of the mean-squared gyration radius $R_{g}$ on time for both dendrimers at temperature T=310 K (the average temperature of a human body) is represented in Figure 2.

Figure 2 demonstrates that the sizes of both dendrimers fluctuate, but their average values and magnitudes of fluctuation do not change with time. It indicates that the dendrimers are close to equilibrium state. The mean square size for Lys-2Lys is close to 2.0 nm and for Lys2Gly it is equal 1.35 nm, thus their ratio is close to 1.5. This result is in agreement with the experimental data [63]. The variation of size of the Lys-2Lys dendrimer occurs between 1.7 nm and 2.3 nm, while the size of the Lys-2Gly dendrimer is in the range of 0.9 to 1.8 nm, i.e., the magnitudes of fluctuations are 0.6 nm and 0.9 nm, respectively. Thus, the relative fluctuations (the ratio of fluctuation to the average size of each dendrimer is equal to 0.6/2.0 = 0.30 and 0.9/1.35 = 0.67, correspondingly). This means that the relative fluctuations of the size for Lys-2Gly dendrimers with 2Gly spacers are more than two times (0.67/0.3) greater than for dendrimers with 2Lys spacers.

**Figure 3:**
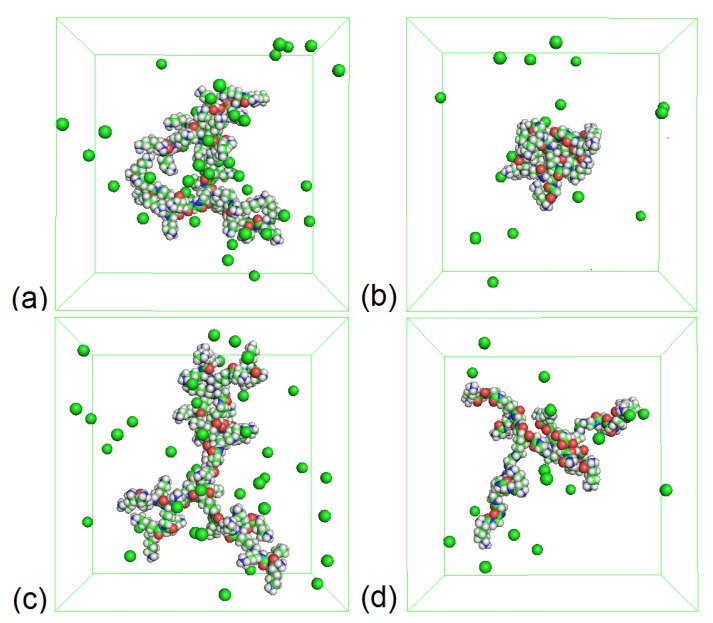
The snapshots of dendrimers at minimal value of $R_{g}$: (**a**) Lys-2Lys (at 69.7 ns, see Figure 2a) and (**b**) Lys-2Gly (at 155.4 ns, see Figure 2b), and at maximal $R_{g}$: (**c**) Lys-2Lys (at 137.3 ns, see Figure 2a) and (**d**) Lys-2Gly (at 78.1 ns, see Figure 2b) at T=310 K.

To demonstrate the differences of fluctuations of the dendrimer sizes, we display in Figure 3 the snapshots of the systems at temperature T=310 K. In Figure 3, the conformations of dendrimers under consideration at minimum ($\min\left[R_{g}\left(t\right)\right]$) and maximum ($\max\left[R_{g}\left(t\right)\right]$) sizes are presented. We can see that Lys-2Gly essentially has a more compact structure than Lys-2Lys in this limit. The conformation of Lys-2Lys at maximum size (Figure 3c) is swollen and extended unlike its conformation at minimal size (Figure 3a). In the case of Lys-2Gly, the conformation maximal size (Figure 3d) is much more swollen in comparison to its conformation at minimal size (Figure 3b). However, Lys-2Gly with 2Gly spacers has an essentially greater conformational freedom than Lys-2Lys with 2Lys spacers. This result has been expected from the results presented in Figure 2.

The timescale of characteristic fluctuations of the dendrimer size as well as of the rotational motion of the dendrimer as a whole can be estimated from the autocorrelation function (ACF) of the square of the gyration radius $R_{g}$,

(2) $$C_{R_{g}^{2}}\left(t\right)\;=\;\frac{\langle R_{g}^{2}\left(\tau \right)\times R_{g}^{2}\left(\tau +t\right)\rangle -{\langle R_{g}^{2}\rangle }^{2}}{\langle R_{g}^{4}\rangle -{\langle R_{g}^{2}\rangle }^{2}}$$

and the first-order orientational autocorrelation function (1st order ACF) for vector from C${}_{\alpha }$ in lysine in the core to the C${}_{\alpha }$ in the terminal lysines:

(3) $$P_{1}^{rot}\left(t\right)\;=\;\langle \frac{\left(r(t),r(0)\right)}{\left|r(t)\right|\left|r(0)\right|}\rangle$$

**Figure 4:**
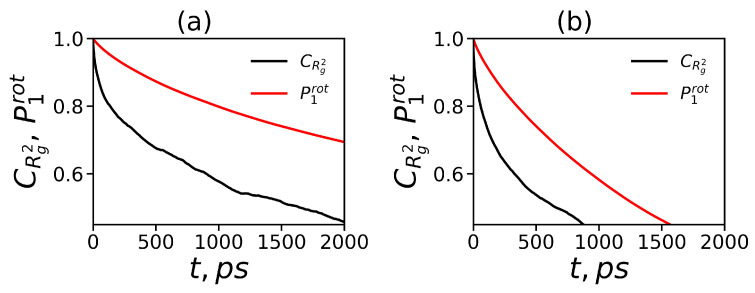
The autocorrelation functions (ACFs): the normalized ACF $C_{R_{g}^{2}}\left(t\right)$ of the square of the gyration radius $R_{g}$ and the orientational ACF $P_{1}^{rot}\left(t\right)$ at T=310 K for (**a**) Lys-2Lys and (**b**) Lys-2Gly.

Examples of the time dependencies of these ACFs are presented in Figure 4 at temperature T=310 K.

According to Figure 4a,b, the orientational ACFs have slower time decays than the ACFs for the square of the dendrimer size. The functions $P_{1}^{rot}\left(t\right)$ and $C_{R_{g}}\left(t\right)$ decay slower for the Lys-2Lys dendrimer than for Lys-2Gly. The characteristic relaxation times $\tau_{rot}^{P_{1}}$ ($P_{1}\left(\tau_{rot}^{P_{1}}\right)=1/e$) obtained at different temperatures from the orientational ACFs are presented in Table 2. These times decrease with an increase of temperature. The values of all times are between 1 to 15 ns, i.e., much smaller than the total time of simulation (250 ns). Thus, the total simulation time is enough to get the well-equilibrated systems. The lysine dendrimers [29] at room temperature have the rotation of a dendrimer as a whole equal to 2.4 ns that it is comparable with the $\tau_{rot}^{P_{1}}\;=\;2.8$ ns for Lys-2Gly and more than 3 times lower than $\tau_{rot}^{P_{1}}$ for Lys-2Lys. The characteristic times for Lys-2Lys at all temperatures are greater than for Lys-2Gly. This result confirms that the Lys-2Gly is more compact at all temperatures than Lys-2Lys.

**Figure 5:**
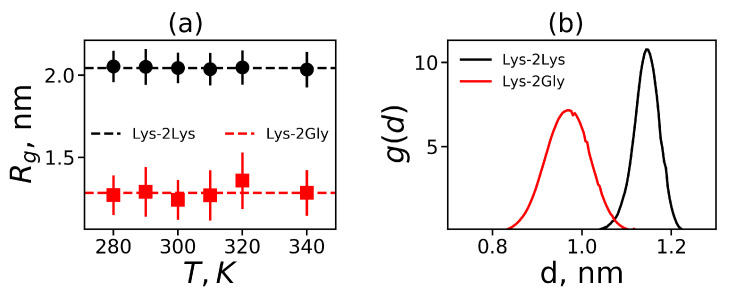
(**a**) The mean square gyration radius $R_{g}$ as a function of temperature; (**b**) the distribution g(d) of a spacer length *d* between the neighboring branching points of the dendrimers at the average body temperature for both dendrimers.

To study the temperature dependences of dendrimer size for both dendrimers we plotted in Figure 5a the gyration radius $R_{g}$ for Lys-2Lys and Lys-2Gly dendrimers as a function of temperature. According to Figure 5a, the gyration radius $R_{g}$ does not change with temperature for both dendrimers. The average values of $R_{g}$ for these dendrimers are depicted by dashed lines in Figure 5a. The size of Lys-2Lys ($R_{g}\approx 2$ nm) is approximately 1.5 times larger than the size of Lys-2Gly ($R_{g}\approx 1.35$ nm). The size of Lys-2Gly is also close to the size of the usual (without insertion of 2Lys or 2Gly spacers) Lys-dendrimer ($R_{g}\;=\;1.2$ nm) simulated in the recent paper [31].

Thus, despite the same contour lengths of paths from the first branching point to the terminal lysines of both dendrimers, the Lys-2Lys is much more swollen at all temperatures than the Lys-2Gly dendrimer. These paths consist of spacers between the neighboring branching points. In our dendrimers of the second generation there are 2Lys or 2Gly spacers inserted into the 0th, 1st, and 2nd subgenerations. We plotted in Figure 5b the distribution g(d) of all these spacer mean square end-to-end distances *d* averaged through all subgenerations for each dendrimer. It is easy to see that 2Gly spacers in Lys-2Gly have a maximum of g(d) at smaller *d*, but its distribution is wider, in agreement with the results of Figure 2. This means that the differences between the gyration radii of both dendrimers can be explained by the differences of the spacer characteristics: the mean square lengths and the magnitudes of length fluctuations. The last characteristic is connected with the flexibility of the spacers. This result is in agreement with the smaller characteristic ratio for the end-to-end distances [91] and the bending energies [92] of linear polyglycine in comparison with linear polylysine peptides. Moreover, this is confirmed by the wide use of short flexible glycine linkers [93] for chemical attaching of the bioactive molecules to nanocontainers (linear peptides and dendrimers) for delivery to the target cells or organs.

Such a difference in the values and fluctuations of the dendrimer sizes, as well as in the spacer lengths might indicate that these dendrimers have different shapes. Moreover, the local images of dendrimers with the smallest and largest $R_{g}$ (see Figure 3) also demonstrate that dendrimers in these states have different shapes. To check the average shape of our dendrimers, we can use the approximation of the dendrimer as a 3D ellipsoid with semi-axes corresponding to eigenvalues $I_{x}$, $I_{y}$, and $I_{z}$ of the dendrimer’s gyration tensor [94]:

(4) $$A_{\mu \nu }\;=\;\frac{1}{N}\left[\sum_{i=1}^{N}\left(\mu_{i}-\mu_{c}\right)\left(\nu_{i}-\nu_{c}\right)\right],\;\mu ,\;\nu \;=\;x,y,z$$

where ($x_{i}$, $y_{i}$, $z_{i}$) and ($x_{c}$, $y_{c}$, $z_{c}$) are coordinates of the *i*-th bead and the center of mass, respectively. The main axes of the tensor allow calculation of the asphericity parameter α [95,96,97]:

(5) $$\alpha \;=\;1-3\frac{I_{x}I_{y}+I_{x}I_{z}+I_{y}I_{z}}{{\left(I_{x}+I_{y}+I_{z}\right)}^{2}}$$

**Figure 6:**
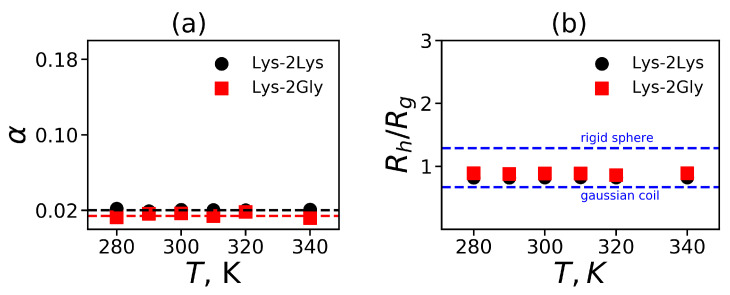
(**a**) The shape anisotropy α and (**b**) the characteristic ratio of hydrodynamic radius in Kirkwood approximation to the gyration radius $R_{h}/R_{g}$; two theoretical limits are depicted on the graph: the Gaussian coil and the rigid sphere.

The value of α is between 0 (for a sphere) and 1 (an extremely elongated ellipsoid). Simulation of different dendrimers shows [95,98,99] that the dendrimers of small generations have an ellipsoidal shape, but their shape becomes more and more spherical with increasing generation number. Figure 6a shows the average value of α as a function of the temperature *T* for Lys-2Lys and Lys-2Gly dendrimers. It is easy to see that α is almost constant and very similar for both dendrimers. The small values of α (~0.02) indicate that these dendrimers have almost spherical shapes. Interestingly, the lysine dendrimer of the same generation without insertion of 2Lys or 2Gly spacers at room temperature [31] has α~0.2, i.e., it is an order of magnitude larger than the values obtained in this study for Lys-2Lys and Lys-2Gly dendrimers.

As our dendrimers have a spherical shape, we can apply to them different theories elaborated for spherical molecules. In particular, we can calculate for our dendrimers the hydrodynamic radius $R_{h}$, the ratio $R_{h}/R_{g}$, and radial density profile. The values of $R_{h}/R_{g}$ are different for different models. For example, the Gaussian coil (the penetrable sphere) model [100] has the lowest value of $R_{h}/R_{g}\;=\;0.67$. The upper limit case is the impenetrable sphere (the homogeneous rigid sphere) with value $R_{h}/R_{g}\;=\;1.29$ [100]. To calculate the hydrodynamic radius of our dendrimers $R_{h}$ from MD simulation we can use the Kirkwood approximation [101,102]:

(6) $$R_{h}^{-1}\;=\;{\langle r_{ij}^{-1}\rangle }_{i\neq j},$$

where $r_{ij}$ is the distance between two atoms *i* and *j*. The ratio $R_{h}/R_{g}$ as a function from temperature is shown in Figure 6b for both dendrimers. The two limiting cases (for the Gaussian coil and for the rigid sphere) are shown by dashed lines too. We evaluated the ratio $R_{h}/R_{g}$ and obtained that it was equal to 0.83 for Lys-2Lys and 0.89 for Lys-2Gly dendrimers at T=310 K (see Table 3). Both values were between theoretical boundaries for penetrable and rigid spheres. This result is in agreement with the experimental study of the ratio $R_{h}/R_{g}$ for usual lysine dendrimers (without insertion of 2Lys or 2Gly spacers) of different generations in dimethylformamide solvent [25]. We would like to note that the ratio $R_{h}/R_{g}$ should increase with an increasing number of generations [25,32] as dendrimer behavior will approach the behavior of a rigid sphere.

The three characteristic sizes of a dendrimer (see Table 3) at T=310 K (the average body temperature) are $\sqrt{5/3}R_{g}$ (in approximation a dendrimer as the rigid sphere), and $R_{e}$ is the mean square distance $r_{i}$ from the ends to the center of mass of the dendrimer:

(7) $$R_{e}\;=\;{\left(\frac{1}{N_{t}}\sum_{i=1}^{N_{t}}r_{i}^{2}\right)}^{1/2},$$

and the effective radius $R_{max}$ (will be defined below). For both dendrimers, the values of $R_{max}$ are larger than other characteristic sizes. For Lys-2Lys, the $\sqrt{5/3}R_{g}$ is close to $R_{e}$, which is a very usual situation for dendrimers. In the case of Lys-2Gly, the $R_{e}$ is larger than the $\sqrt{5/3}R_{g}$, meaning that the terminal groups are more often located on the periphery of the dendrimer.

### 3.2. The Local Structure

**Figure 7:**
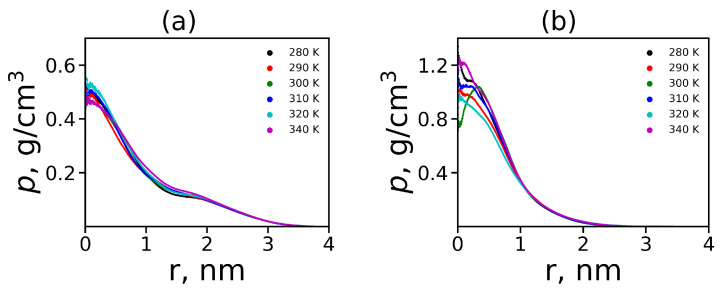
The radial distribution function of density for (**a**) Lys-2Lys and (**b**) Lys-2Gly dendrimers at different temperatures.

We have shown earlier that both dendrimers have a spherical shape. For such objects the local internal structure is well described by the radial density distribution function (RDF) of atoms relative to the center of mass of the dendrimer (a density profile) ρ(r):

(8) $$\rho \left(r\right)\;=\;\frac{1}{4\pi r^{2}}\sum_{i}m_{i}\delta \left(r-r_{i}\right)$$

where $r\;(r_{i})$ is the distance (from *i*-th atom) to the center of mass. The density profiles are shown in Figure 7. The radial distributions for Lys-2Lys are practically independent of temperature. For Lys-2Gly, the temperature affects the density profile in the area of the dendrimer center, but does not affect the overall shape of RDF. In general, the Lys-2Lys dendrimer has a looser structure and a density in the center about two times (0.5 instead of 1.0) less than the Lys-2Gly dendrimer. However, the decrease in density with distance *r* from the center in the Lys-2Lys dendrimer does not occur quickly as in the Lys-2Gly dendrimer. This behavior correlates with a larger size of the Lys-2Lys dendrimer in contrast to the Lys-2Gly dendrimer. The shape of the density profile of the Lys-2Gly dendrimer is also close to that one of the usual lysine dendrimer [31].

**Figure 8:**
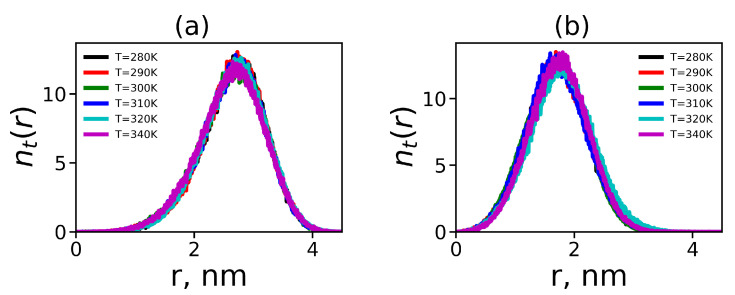
The radial distribution of the number of N atoms in terminal NH${}_{3}$+ groups for (**a**) Lys-2Lys dendrimer and (**b**) Lys-2Gly dendrimer.

In each terminal lysine, marked by red color in Figure 1, there are two charged NH${}_{3}$+ terminal groups. The radial distributions of the number $n_{t}\left(r\right)$ of these nitrogens in all terminal groups for Lys-2Lys and Lys-2Gly dendrimers at different temperatures are shown in Figure 8. This function for both dendrimers is practically independent of temperature. The radial distribution $n_{t}\left(r\right)$ for Lys-2Lys (see Figure 8a) has an asymmetric shape and is shifted to the right (to larger distances *r* from the center of mass of the dendrimer) compared to the distribution for Lys-2Gly that has (see Figure 8b) an almost symmetrical shape. The shape of $n_{t}\left(r\right)$ for Lys-2Gly is close to the shape for the usual lysine dendrimer at room temperature [31]. The positions of maxima correspond to the distances between the core and the terminal groups $R_{e}$ in these dendrimers (see Table 3).

The density profiles and the radial distributions of terminal nitrogens for these dendrimers confirm that the Lys-2Lys dendrimer is swollen and stretched more than the Lys-2Gly dendrimer. It is due to the strong electrostatic interactions of the charged 2Lys spacers inside the Lys-2Lys dendrimer.

**Figure 9:**
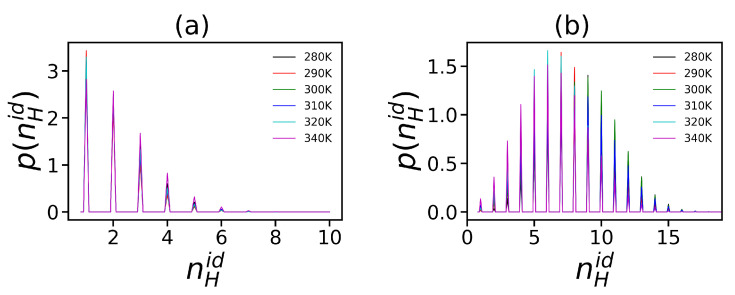
The distribution function of the number of intra-dendrimer hydrogen bonds at different temperatures for (**a**) Lys-2Lys and (**b**) Lys-2Gly dendrimers.

Hydrogen bonds exist between the donor and acceptor groups [103,104,105,106]. The presence of hydrogen bonds is determined by simulation using the coordinates of donor and acceptor according to two rules: the maximum distance between the donor and the acceptor should be less than 0.35 nm, and the angle between the donor and acceptor bonds should be less than 30° [106]. The average distribution of the number of intra-dendrimer hydrogen bonds is depicted in Figure 9.

We obtained that these distributions had a subtle dependence on temperature for both dendrimers. At the same time, there is an essential difference between the shape of these functions for our dendrimers at all temperatures. For Lys-2Lys, it is a decreasing function, while for Lys-2Gly it has a fairly symmetrical shape. The average number of hydrogen bonds is less than two for Lys-2Lys, in comparison with more than seven bonds for Lys-2Gly (see Table 4). This difference can be explained by the fact that Lys-2Lys is more swollen, stretched, and rigid than the compact and flexible Lys-2Gly dendrimer. Therefore, it is much more difficult for donors and acceptors in Lys-2Lys to meet each other and form a hydrogen bond. Moreover, the average number of dendrimer–water hydrogen bonds (see Table 4) is essentially larger in Lys-2Lys in comparison with Lys-2Gly, equal to 179 and 100, respectively.

**Figure 10:**
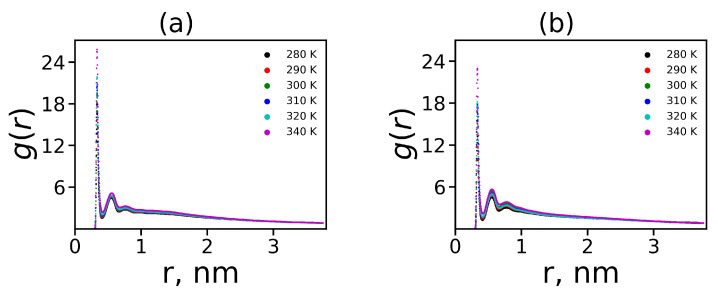
The radial distribution function of ion pairs Cl${}^{-}$-N${}^{+}$ at different temperatures for lysine-based dendrimers (**a**) Lys-2Lys and (**b**) Lys-2Gly.

It is important to note that electrostatic interactions contribute to the formation of ion pairs. The interactions between charged groups of dendrimer (NH${}_{3}^{+}$) and counterions can be characterized by the binary distribution function g(r) for distance *r* between these ion pairs. Figure 10 shows these characteristics for both dendrimers at different temperature. It is easy to see that there is some dependence on the temperature of the value of the first peak g(r), but the overall shape of this dependence almost does not change with temperature. The first peak corresponds to the minimal distance between the charged groups of the dendrimer and ions, i.e., this region corresponds to the formation of ion pairs. Lys-2Lys has a slightly higher peak than Lys-2Gly. This means that Lys-2Lys has more ion pairs than Lys-2Gly because the first system has more charges and counterions. We estimated the number of ion pairs $\langle n_{ion\;pairs}\rangle$ more accurately by numerical integration over the region of the first peak (see Table 4). The number of ion pairs in Lys-2Lys is on average 8 times larger than in Lys-2Gly. The second peak corresponds to the distance between counterions and the neighboring oppositely charged groups of the dendrimer.

In addition, the counterions come into contact with the dendrimer trying to lower their bare charge $Q_{bare}$. To evaluate how the total charge is distributed in the system due to the redistribution of counterions between the solution and the dendrimer interior, we can calculate the radial distribution of the total charge q(r) (the charge distribution) in the system:

(9) $$q\left(r\right)\;=\;n_{+}\left(r\right)+n_{-}\left(r\right)$$

where $n_{+}\left(r\right)$ is the positive charge distribution of NH${}_{3}^{+}$-groups of dendrimers and $n_{-}\left(r\right)$ is negative charge distribution of counterions. The integration of the charge distribution q(r) over the simulation cell is equal to zero, confirming the electroneutrality of the simulation system.

**Figure 11:**
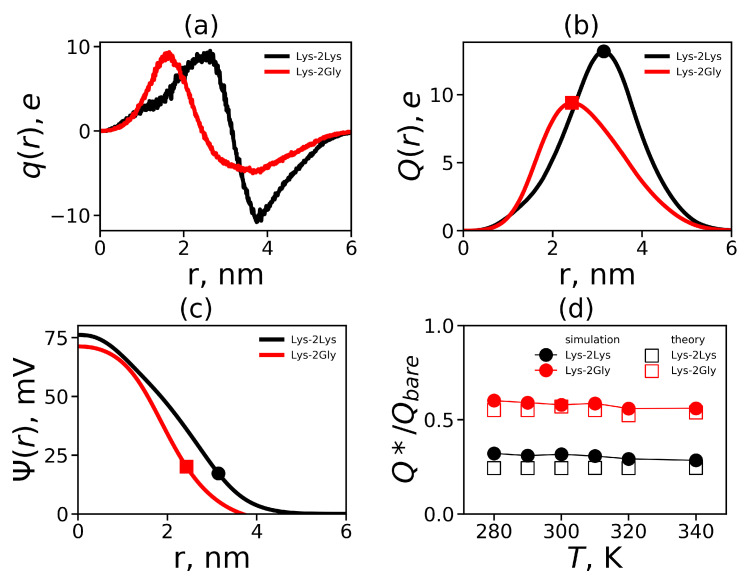
The radial distribution of (**a**) the total charge q(r), (**b**) the cumulative charge Q(r), and (**c**) the electrostatic potential at T=310 K for both dendrimers. (**d**) The relative effective charge $Q*/Q_{bare}$ as a function of temperatures for the Lys-2Lys and the Lys-2Gly dendrimers.

The charge distribution q(r) is plotted in Figure 11a for the Lys-2Lys and the Lys-2Gly dendrimers. This function for both dendrimers has a maximum due to the positive charges of the dendrimers and a minimum due to the counterion shells around them. The charge distribution obtained in our simulations corresponds to the classical double layer structure, which has been observed for dendrimers in many studies [107,108]. It is interesting that the values in maximum are approximately the same in the case of Lys-2Lys and Lys-2Gly dendrimers. At the same time, the positions of the maximum and minimum are shifted to greater radial distance *r* for Lys-2Lys dendrimer.

The cumulative charge distribution Q(r) is the integral characteristic of the charge distribution q(r). It can be calculated from Equation (10), where the integral has the variable upper limit

(10) $$Q\left(r\right)\;=\;\int_{0}^{r}q\left(x\right)dx$$

The cumulative charge in layer *r* corresponds to the total charge from the center of the system to the layer *r*. The cumulative charge distributions Q(r) for the considered dendrimers are depicted in Figure 11b. For Lys-2Lys, the absolute value of the maximum is larger, and its position is more shifted from the center of mass compared to for Lys-2Gly. The maxima of these distributions correspond to the effective charge Q*, and the positions of maxima (marked with dots in Figure 11b) correspond to the effective radius $R_{max}$. In general, the distribution Q(r) for Lys-2Gly is very similar in shape, position, and the value of maximum to the usual lysine dendrimer [31].

Our dendrimers can be represented as charged macroions. To evaluate the electrostatic interactions inside and outside such a system, we can consider the distribution of the electrostatic potential Ψ(r), which can be calculated from the Poisson equation

(11) Δψ=−kq(r),

Here, $\psi \left(r\right)\;=\;\left[e/k_{B}T\right]\Psi \left(r\right)$ is the dimensionless electrostatic potential, and $k\;=\;4\pi \lambda_{B}/dr$ is the dimensionless factor (dr [nm] is *r* increment), where $\lambda_{B}$ is the Bjerrum length (≈0.7 nm for the water medium at room temperature):

(12) $$\lambda_{B}\;=\;\frac{e^{2}}{4\pi \epsilon \epsilon_{0}k_{B}T}$$

where *e* is the elementary charge, ϵ is the relative dielectric permittivity of water (ϵ≈80), $\epsilon_{0}$ is the dielectric permittivity of vacuum, $k_{B}$ is the Boltzmann constant, and *T* is the actual temperature.

In the case of the spherical symmetry, the Poisson equation (Equation (11)) can be presented in a simple form of the differential equation

(13) $$\frac{d^{2}\psi \left(r\right)}{dr^{2}}+\frac{2}{r}\frac{d\psi (r)}{dr}\;=\;-kq\left(r\right)$$

This equation can be solved numerically, for example, by the method of successive approximations.

To solve Equation (13), we used boundary condition ψ(D)=0 (the electrostatic potential is zero in the simulation cell edge). Moreover, we plotted the electrostatic potentials for both dendrimers at T=310 K in Figure 11c. The points on the curves indicate the effective radius $R_{max}$, after which the diffusion layer begins. We have used this approximation to estimate the ζ potential, which works quite well in the free-salt case [109]. The distribution of ψ(r) for Lys-2Lys is on average higher than that for Lys-2Gly, because Lys-2Gly has a smaller effective radius $R_{max}$. The value of the ζ potential for the last one is higher (see Table 4). The ζ potential for the lower values (less 25.4 mV [110]) is proportional to the surface charge density $\sigma \;=\;Q*/4\pi R_{max}^{2}$ [110]. In other words, the lower value of σ corresponds to the lower value of the zeta potential (see Table 4).

It is possible to calculate the degree of a recharge dendrimer due to the penetration of counterions into the dendrimer interior using the ratio $Q*/Q_{bare}$. As the considered dendrimers have a spherical shape, they can be represented like soft charged (penetrable) spheres (charged macroions). The ratio $Q*/Q_{bare}$ can be calculated from the theoretical considerations. We used the analytical theory [111] for calculation an effective charge Q* of the soft charged sphere with the radius *R* and the bare charge *Q*:

(14) $$Q*\;=\;\frac{R}{\lambda_{B}}\frac{1}{2\nu }\ln\left[\left(\frac{Q}{Q*}-1\right)\left(\frac{D^{3}}{R^{3}}-1\right)\right]$$

where the coefficient ν=3/5 (for a diluted solution). As mentioned above, the Lys-2Lys and Lys-2Gly have spherical shapes (see Figure 6a) and can be considered as charged spheres with a radius of $R\;=\;R_{max}$ (the position of the maximum of the cumulative charge distribution Q(r)) and a bare charge of $Q_{bare}$ (see Table 1). Then, the radius $D\;=\;3^{1/2}a_{cell}/2$, where $a_{cell}$ is the average size of a simulation cell (see Table 1).

Figure 11d demonstrates the ratios $Q*/Q_{bare}$ obtained using the theoretical predictions for Q* and also the values $Q*/Q_{bare}$ obtained from simulation using the maximum of the cumulative charge $Q*\;=\;Q_{max}$ as a function of temperature. Thus, the simulation results are close to the theoretical predictions in Equation (14). A similar good agreement was obtained in MD simulation of usual lysine dendrimer [31]. The ratio $Q*/Q_{bare}$ is the same for the Lys-2Gly and the usual lysine dendrimers of the second generation at T=300 K [31]. The osmotic ions do not form ion pairs with charged groups of a dendrimer. Moreover, they are freely distributed in the dendrimer interior causing osmotic pressure, which leads to the stretching the dendrimer. The average number of osmotic ions $n_{osmotic\;ions}$ can be calculated from the following equation.

(15) $$n_{osmotic\;ions}\;=\;Q_{bare}-Q*-\langle n_{ion\;pairs}\rangle$$

The average numbers of osmotic ions $n_{osmotic\;ions}$ at T=310 K were equal to 25.13 for Lys-2Lys and 5.94 for Lys-2Gly (Table 4). In other words, the number of free counterions inside Lys-2Lys is 4.2 times larger than inside Lys-2Gly.

### 3.3. Orientational Mobility and NMR Relaxation

**Figure 12:**
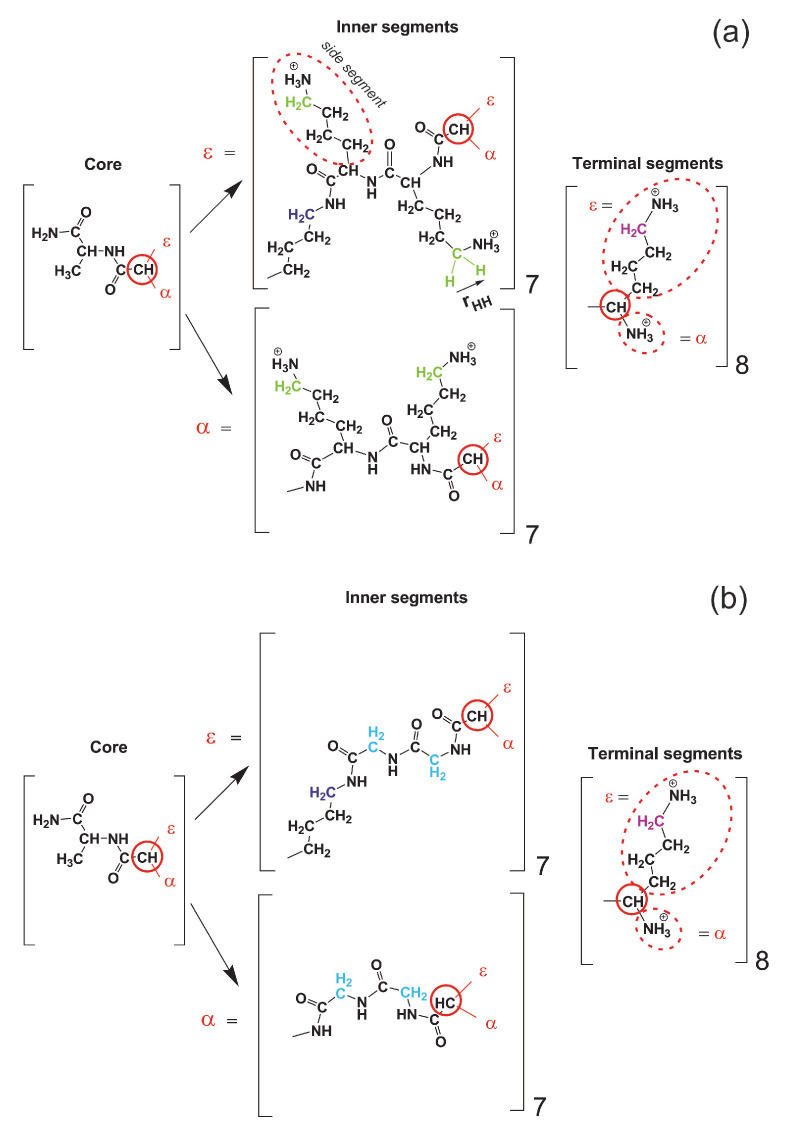
The structures of (**a**) the Lys-2Lys and (**b**) the Lys-2Gly dendrimers. Red circles show the CH-branching points in the core, inner, and terminal segments. The inner and terminal CH${}_{2}$ groups are marked in blue and magenta colors, respectively. In Lys-2Lys, there are CH${}_{2}$ groups in side segments of 2Lys spacers marked in green. In Lys-2Gly, there are CH${}_{2}$ groups in each inserted 2Gly spacer marked by light blue. HH vector ($r_{HH}$) is shown for one CH${}_{2}$ group.

In this section, we describe the results of the calculations of orientational mobility of different vectors inside each dendrimer including core-to-end vectors (averaged over all paths from the center of the dendrimer to its ends) and HH vectors in different CH${}_{2}$ groups. Two main types of CH${}_{2}$ groups were considered: (1) the CH${}_{2}$ groups connected with NH groups are in the backbone and named as “inner” CH${}_{2}$ groups; (2) the CH${}_{2}$ groups are connected with NH${}_{3}^{+}$ groups are in the terminal segments and named as “terminal” CH${}_{2}$ groups. In Lys-2Lys there are also additional CH${}_{2}$ groups connected with NH${}_{3}^{+}$ groups in side segments of 2Lys spacers (marked in green in Figure 12a), and we named them as “side” CH${}_{2}$ groups. In Lys-2Gly there are two CH${}_{2}$ groups in the main chain of each inserted 2Gly spacer (marked by light blue in Figure 12b), and we named them as “inner-Gly” CH${}_{2}$ groups.

The rotation of the dendrimer as a whole is usually characterized through the mobility of the core-to-ends vector, which can be estimated from the second-order ACF:

(16) $$P_{2}\left(t\right)\;=\;\frac{3}{2}\langle \frac{{\left(r(t)r(0)\right)}^{2}}{{\left|r(t)\right|}^{2}{\left|r(0)\right|}^{2}}\rangle -\frac{1}{2}$$

Here, we use core-to-end vectors that started in the Cα atom of core lysine to Cα atom in terminal lysines. The averaging $\langle \times \rangle$ in the Equation (16) means averaging over all core-to-end vectors and over considered time.

There is a simple relationship [112] between the 1st order and 2nd order ACFs for a single rigid bond:

(17) $$P_{2}\left(t\right)\;=\;{\left(P_{1}\left(t\right)\right)}^{3}$$

**Figure 13:**
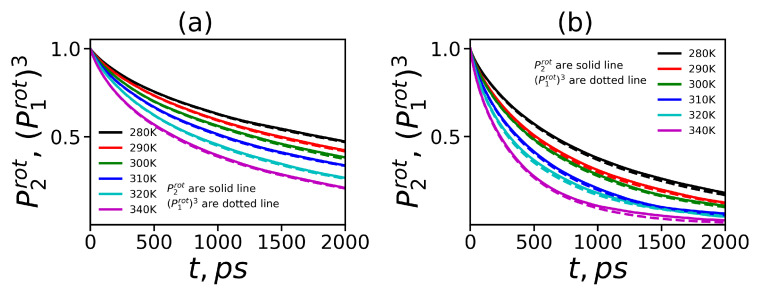
The autocorrelation functions $P_{2}^{rot}$ and ${\left(P_{1}^{rot}\right)}^{3}$ for the core-to-end vector of (**a**) Lys-2Lys and (**b**) Lys-2Gly dendrimers.

In several papers on the simulation of dendrimers (see, for example, in [29]) it has been shown that this equation is also valid for the orientational mobility of dendrimer as a whole. Here, we examine the relation Equation (17) for the core-to-end vectors of our dendrimers. Figure 13a,b demonstrates that this relation is valid for both dendrimers at all temperatures. Using the relationship in Equation (17) we evaluated the characteristic time of $P_{2}^{rot}\left(t\right)$:

(18) $$\tau_{rot}\;=\;\frac{\tau_{rot}^{P_{1}}}{3}$$

**Figure 14:**
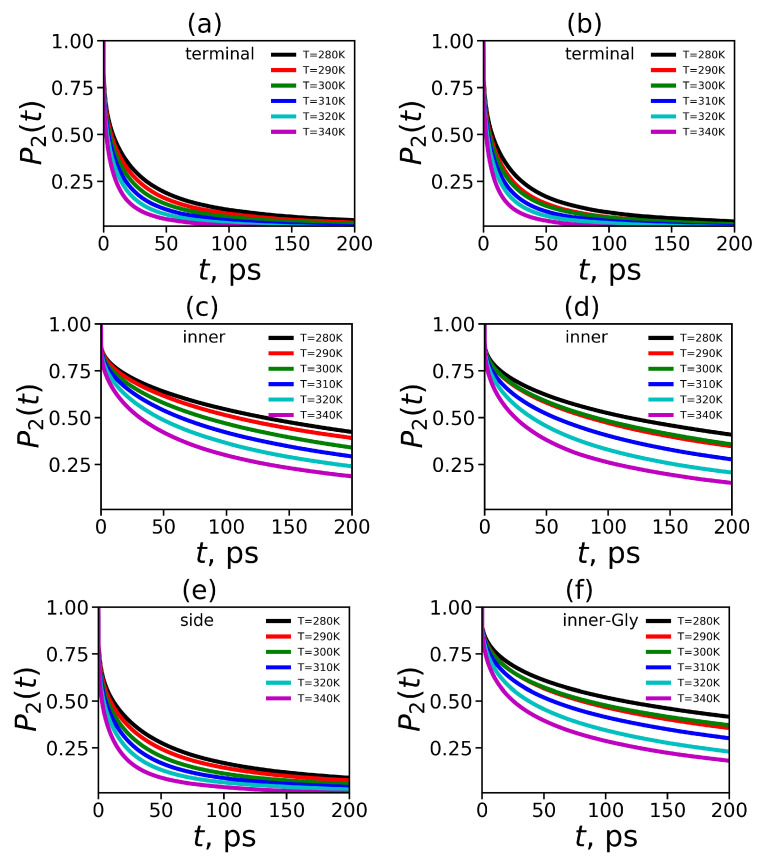
The time dependencies of $P_{2}\left(t\right)$ for terminal CH${}_{2}$ groups of (**a**) Lys-2Lys and (**b**) Lys-2Gly, for the inner CH${}_{2}$ group of (**c**) Lys-2Lys and (**d**) Lys-2Gly, (**e**) for the side CH${}_{2}$ group of Lys-2Lys, and (**f**) for inner-Gly CH${}_{2}$ groups of Lys-2Gly.

To obtain characteristics of NMR relaxation of CH${}_{2}$ groups in our dendrimers we have computed the second-order autocorrelation function $P_{2}\left(t\right)$ (see Equation (16)) for vector $r=r_{HH}$ in different CH${}_{2}$ groups. We obtained that the decay of this function for both dendrimers is similar for terminal groups (see Figure 14a,b) as well as for inner groups (see Figure 14c,d). At the same time, the ACF or terminal CH${}_{2}$ groups decays significantly faster than that of the inner CH${}_{2}$ groups for both dendrimers (see Figure 14a,c vs. Figure 14b,d). This result is in agreement with the prediction of theory for dendrimer molecules [113]. Moreover, we calculated the orientational ACF for side CH${}_{2}$ groups of the Lys-2Lys dendrimer (see Figure 14e) and for inner-Gly CH${}_{2}$ groups of the Lys-2Gly dendrimer (see Figure 14f). The behavior of the $P_{2}$ function for the side CH${}_{2}$ groups is very similar to the behavior of the terminal groups of Lys-2Lys (Figure 14a,e). In contrast, the relaxation of the inner-Gly CH${}_{2}$ groups (see Figure 14f) is similar to relaxation of the inner CH${}_{2}$ groups (see Figure 14b) of the Lys-2Gly dendrimer.

The reduced spin-lattice relaxation time $\left[1/T_{1H}\right]$ in the susceptibility representation is one of the characteristics of the mobility of groups. This parameter is connected with the spectral densities J(ω) and J(2ω) by the following relation [114,115],

(19) $$\left[\frac{1}{T_{1H}}\right]\left(\omega \right)\;=\;\omega \left(J\left(\omega \right)+4J\left(2\omega \right)\right)$$

where ω is the angular frequency of NMR spectrometer; the spectral density $J\left(\omega \right)$ can be calculated from the cosine Fourier transformation:

(20) $$J\left(\omega \right)\;=\;2\int_{0}^{\infty }P_{2}\left(t\right)\cos\left(\omega t\right)dt$$

**Figure 15:**
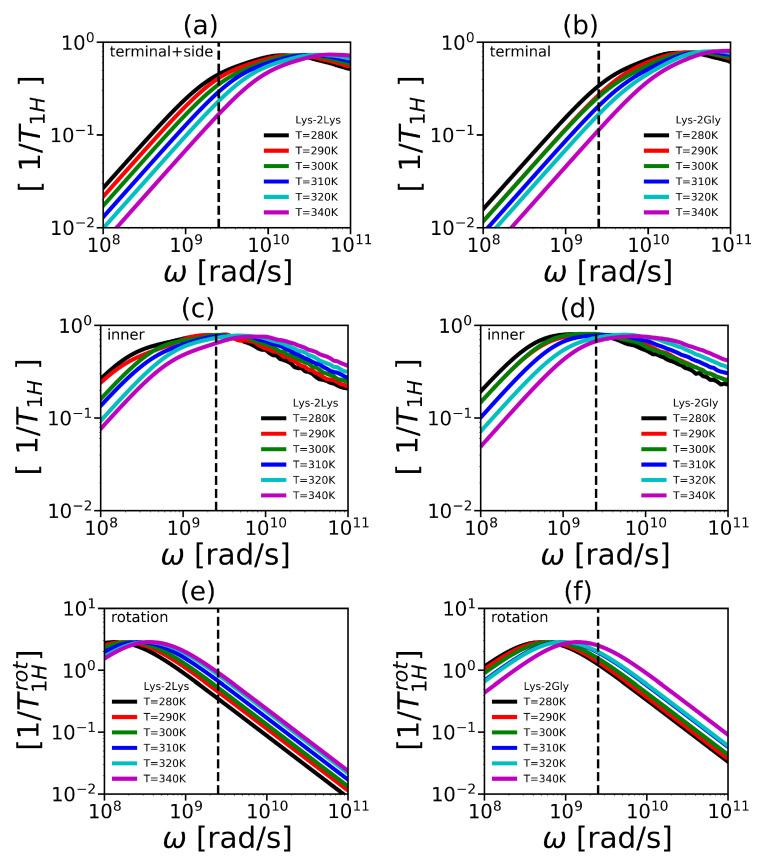
Frequency dependencies of $\left[1/T_{1H}\right]$ in the susceptibility representation of (**a**) terminal+side CH${}_{2}$ groups in Lys-2Lys, (**b**) terminal CH${}_{2}$ groups in Lys-2Lys Lys-2Gly, inner CH${}_{2}$ groups in (**c**) Lys-2Lys and (**d**) Lys-2Gly, and the core-to-end vector in (**e**) Lys-2Lys and (**f**) Lys-2Gly. The vertical line represents to the frequency ($\omega_{H}/2\pi \;=\;400$ MHz), which corresponds to the spectrometer used in [63].

The reduced spin-lattice relaxation rate $\left[1/T_{1H}\right]$ is shown in Figure 15. We combined the side and terminal groups in Lys-2Lys into one group—“terminal+side” (see Figure 15a) as a similar procedure was used in NMR [63]. Furthermore, there was not a significant difference between them in our simulation. The frequency dependencies of $\left[1/T_{1H}\right]$ for the terminal group for Lys-2Gly (see Figure 15b) and inner groups of both dendrimers (see Figure 15c,d) are presented in Figure 15.

According to Equations (19) and (20), the $\left[1/T_{1H}\right]$ functions can be computed through ACFs $P_{2}\left(t\right)$. Note that the numerical procedure for the calculation of ACFs $P_{2}\left(t\right)$ functions is described in Appendix [29]. The resulting function was combined with two parts: $1\leq P_{2}\left(t\right)\leq P_{cut}$ ($P_{cut}\;=\;$ 0.04, 0.05, 0.05, and 0.08 for terminal, side, inner-Gly, and inner groups, respectively) and $P_{cut}\exp\left(-\left(t-\tau_{tail}\right)/\tau_{tail}\right)$. Here, $\tau_{tail}$ is determined from the exponential slope of the $P_{2}\left(t\right)$ function at $n_{approx}$ points ($n_{approx}$ = 100, 100, 100, and 1000 for the terminal, side, inner-Gly, and inner groups, respectively) to the value $P_{cut}$ by least square method. We have ensured that the resulting function decays to 1 × 10${}^{-5}$.

To estimate the contribution of rotation of a dendrimer as a whole to the mobility of CH${}_{2}$ groups we also calculated the $\left[1/T_{1H}^{rot}\right]$ function for the core-to-end vector. $P_{2}^{rot}\left(t\right)$ is the linear combination of the decreasing exponential functions. Due to the rotation of a dendrimer as a whole, $\tau_{rot}$ makes the dominant contribution, and we can use the single exponential approximation for calculation of $P_{2}^{rot}\left(t\right)$:

(21) $$P_{2}^{rot}\left(t\right)\approx \exp\left(-\frac{t}{\tau_{rot}}\right)$$

The cosine Fourier transformation (see Equation (20)) of $P_{2}^{rot}\left(t\right)$ has a simple form:

(22) $$J^{rot}\left(\omega \right)\;=\;2\times \frac{\tau_{rot}}{1+{\left(\omega \tau_{rot}\right)}^{2}}$$

and the reduced spin-lattice relaxation time $\left[1/T_{1H}^{rot}\right]$ for the core-to-end vector can be expressed as

(23) $$\left[1/T_{1H}^{rot}\right]\left(\omega \right)\;=\;2\omega \left[\frac{\tau_{rot}}{1+{\left(\omega \tau_{rot}\right)}^{2}}+4\times \frac{\tau_{rot}}{1+4{\left(\omega \tau_{rot}\right)}^{2}}\right]$$

**Figure 16:**
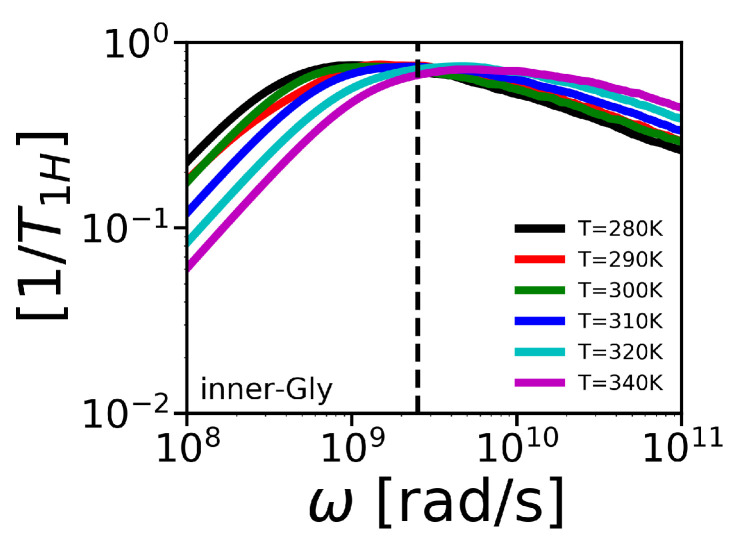
Frequency dependencies of $\left[1/T_{1H}\right]$ of inner-Gly CH${}_{2}$ groups in Lys-2Gly. The vertical line corresponds to the frequency $\omega_{H}/2\pi \;=\;400$ MHz of the spectrometer used in [63].

Note that this result for $\left[1/T_{1H}^{rot}\right]$ is valid if the rotation of a dendrimer as a whole is the only one process contributing to NMR relaxation. The frequency dependencies of $\left[1/T_{1H}^{rot}\right]$ functions are plotted in Figure 15e,f. The mobility of inner-Gly CH${}_{2}$ group is presented in Figure 16.

The important parameter of $\left[1/T_{1H}\right]$ is the position of the maximum $\omega_{max}$. An increase in temperature shifts the position of $\omega_{max}$ to the low-frequency region. It means an acceleration of orientational mobility with temperature. The positions of maxima of $\left[1/T_{1H}\right]$ allow us to compare the mobility of different groups. For terminal CH${}_{2}$ groups of both dendrimers, $\omega_{max}$ are located at higher frequencies in comparison the positions of $\omega_{max}$ for inner CH${}_{2}$ groups. It demonstrates that inner groups have lower mobility than the terminal groups. This fact is typical for dendrimers [113] including lysine dendrimers [29]. Note that the positions of $\omega_{max}$ are similar for the Lys-2Lys and the Lys-2Gly dendrimers. Interestingly, the rotation of a dendrimer as a whole has significantly different relaxation time for both dendrimers (see Table 2). In Figure 15e,f, the function $\left[1/T_{1H}^{rot}\right]$ is presented for illustration. It can be seen from Figure 15 that the $\omega_{max}$ of $\left[1/T_{1H}^{rot}\right]$ is close to $\omega_{max}$ of $\left[1/T_{1H}\right]$ for inner CH${}_{2}$ groups of Lys-2Gly. Therefore, we suppose that the rotation of a dendrimer as a whole makes the main contribution to NMR relaxation of the inner groups of Lys-2Gly. It should be noted that a similar result has been recently obtained for poly(propylene imine) dendrimers in a compact conformation (i.e., similar to Lys-2Gly) by MD simulations [116]. It was shown that the position of $\omega_{max}$ practically coincides with the rotation of a dendrimer as a whole for all inner segments.

**Figure 17:**
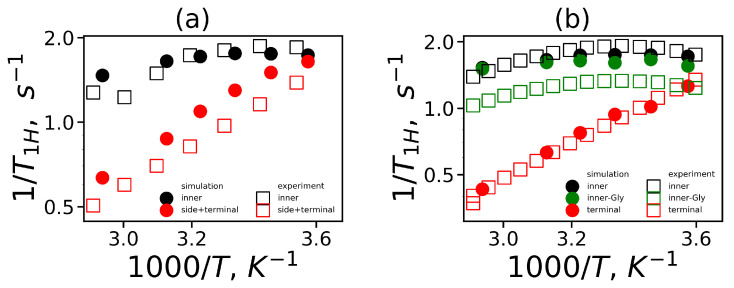
The spin-lattice ${}^{1}$H NMR relaxation rate $1/T_{1H}$ at the fixed frequency $\omega_{H}/2\pi \;=\;400$ MHz as a function of inverse temperature 1000/T for (**a**) Lys-2Lys and (**b**) Lys-2Gly dendrimers calculated from MD simulation. The experimental points [63] are also depicted in the graph.

The frequency dependencies of $\left[1/T_{1H}\right]$ at different temperatures make it possible to calculate the corresponding temperature dependencies. We extracted temperature points from $\left[1/T_{1H}\right]$ at $\omega_{H}/2\pi \;=\;400$ MHz (see dashed vertical line in Figure 15a–d and Figure 16a,b) to compare with the experimental $1/T_{1H}$, obtained by NMR, using the following equation,

(24) $$\frac{1}{T_{1H}}\;=\;\frac{A_{0}}{\omega_{H}}\left[\frac{1}{T_{1H}}\right]$$

where $A_{0}$ is the constant determined by quantum chemistry parameters and does not depend on frequency or temperature. The theoretical value of $A_{0}$ for CH${}_{2}$ groups $A_{0}^{theory}$ is equal to $0.56\times 10^{10}$ s${}^{-2}$. At the same time, a fitting parameter $A_{0}$ is often used (see, for example, in [29]). For calculation of the temperature dependence of $1/T_{1H}$ we used $A_{0}^{theory}$ for all CH${}_{2}$ groups except CH${}_{2}$ groups in terminal+side groups of Lys-2Lys and terminal groups of Lys-2Gly (for which the value $A_{0}\;=\;0.88\times 10^{10}$ s${}^{-2}$ was used). The temperature dependencies of $1/T_{1H}$ for different CH${}_{2}$ groups in Lys-2Lys and Lys-2Gly dendrimers are plotted in Figure 17a,b.

The experimental points [63] are depicted in these graphs too. As it can be seen from Figure 17, our results have a good agreement with the experiment. It is interesting to note that a calibration parameters are not used to fit the calculation results for inner and inner-Gly CH${}_{2}$ groups with the experimental data. The calibration parameter was used only for CH${}_{2}$ groups in terminal+side groups of Lys-2Lys and terminal groups of Lys-2Gly.

In general, the frequency dependencies of $\left[1/T_{1H}\right]$ and the temperature ones indicate that the terminal and side CH${}_{2}$ groups have higher mobility than the inner CH${}_{2}$ groups. The dependence of $\left[1/T_{1H}\right]$ for inner-Gly groups is similar to $\left[1/T_{1H}\right]$ for inner groups because both CH${}_{2}$ groups located in the backbone of the dendrimer. These results were obtained by simulation and NMR (see Figure 17). Thus, the orientation mobility of the NMR group in the segment between branching points practically does not depend on the location of this group in the segment. This conclusion confirms the possibility of using the coarse-grained models for qualitative study of local orientational mobility in dendrimers. Such models were used in the analytical theory [117,118,119,120,121] and in simulations [33,122,123,124,125].

## 4. Conclusions

Lysine-based dendrimers with double lysine and glycine linear spacers inserted between neighboring branching points of a usual lysine dendrimer were simulated by molecular dynamics method in the wide interval of temperatures (from 280 to 340 K). It was shown that the size and internal structure of both dendrimers practically do not depend on temperature. The Lys-2Lys dendrimer has more stretched spacers, and as a result it has the larger size in comparison with the Lys-2Gly dendrimer. Lys-2Lys has a lower density and contains more water and counterion molecules in its interior. This leads to a greater number of hydrogen bonds between the dendrimer and water as well as to a large number of ion pairs between charged dendrimer groups and counterions. The relative effective charge $Q*/Q_{bare}$ is twice as large in Lys-2Lys than in Lys-2Gly. It is worth noting that the relative charge slightly decreases with temperature, and rearrangements are observed in the inner regions of the dendrimers. The larger surface of the Lys-2Lys dendrimer leads to greater surface charge density σ and lower ζ potential of this dendrimer.

Dynamic characteristics have been studied as well. It has been shown that the Lys-2Lys dendrimer rotates more slowly than Lys-2Gly. At the same time, the local orientational mobility of the CH${}_{2}$(-N) groups in inner and terminal segments in Lys-2Lys is close to the local orientational mobility of those groups in Lys-2Gly, and comparable to the mobility of CH${}_{2}$ groups in a usual lysine dendrimer. The dependencies of the spin-lattice relaxation rate for terminal and inner CH${}_{2}$ groups were compared with each other and with the experimental NMR data. It has been shown that the mobility of terminal groups is essentially larger than the mobility of inner groups. A good agreement between simulation and experimental data for both types of CH${}_{2}$ groups was obtained. However, the calibration parameter $A_{0}$ for $1/T_{1H}$ of terminal groups is larger than its theoretical value. The mobility of inner-Gly CH${}_{2}$ groups was studied also. The spin-lattice relaxation rates of the same groups in both dendrimers are similar, and their values at all temperatures are very close to experimental values.

In general, the large and charged side groups of 2Lys spacers in the Lys-2Lys dendrimer lead to larger size and smaller fluctuations of this dendrimer in comparison with the Lys-2Gly dendrimer, but they almost do not affect the local internal mobility of the same groups in dendrimers.

The synthesis, NMR properties [63], and biomedical applications [57] of peptide dendrimers with Lys2Lys and Lys2Gly repeating units were published by us in recent years. These two dendrimers were chosen because they have a total positive charge large enough (+16 and +44, correspondingly) for complexation with negatively charged DNA and RNA molecules for application in gene delivery. The Lys-2Lys dendrimer has a larger charge which is good for complexation, but it has a rather rigid backbone that could limit its conformational rearrangement in the complexes with RNA or DNA. As the Lys-2Gly dendrimer has a smaller charge, it should have a greater internal conformational mobility because 2Gly spacers between branching points are much more flexible than the rigid 2Lys spacers in the first dendrimer. Moreover, due to this reason, the branches of the Lys-2Gly dendrimer could better rearrange around RNA and DNA molecules providing better contact with them.

In the future, we plan to continue theoretical and experimental studies of these dendrimer structures of the high generations and the peptide dendrimers with other amino acid compositions.

## Figures and Tables

**Table 1:** The characteristics of Lys-2Lys and Lys-2Gly dendrimers: the molecular mass of dendrimer *M* and dendrimer charge $Q_{bare}$, number $N_{end}$ and charge $Q_{end}$ of terminal groups as well as number $N_{ins}$ and charge $Q_{ins}$ of inserted amino acid residues, the total number $N_{H_{2}O}$ of water molecules in systems, and the average size $a_{cell}$ of the simulation cell.

Dendrimer	*M* (g/mol)	$Q_{\mathrm{bare}}$ (e)	$N_{\mathrm{end}}$	$Q_{\mathrm{end}}$ (e)	$N_{\mathrm{ins}}$	$Q_{\mathrm{ins}}$ (e)	$N_{H_{2}0}$	$a_{\mathrm{cell}}$ (nm)
Lys-2Lys	5695.08	+44	16	+16	28	+28	13,228	7.5
Lys-2Gly	3675.44	+16	16	+16	28	0	13,396	7.5

**Table 2:** The characteristic times $\tau_{rot}^{P_{1}}$ (ns) of the rotation of Lys-2Lys and Lys-2Gly dendrimers as a whole.

Temperature	Lys-2Lys	Lys-2Gly
280 K	13.7	3.6
290 K	10.5	3.2
300 K	9.0	2.8
310 K	7.0	2.1
320 K	5.5	2.0
340 K	5.1	1.3

**Table 3:** The global properties of Lys-2Lys and Lys-2Gly dendrimers: the mean square gyration radius $R_{g}$, the hydrodynamic radius $R_{h}$ in Kirkwood approximation, the characteristic ratio $R_{h}/R_{g}$, the approximation $\sqrt{5/3}R_{g}$ of dendrimer boundary, the effective radius $R_{max}$, and the shape anisotropy α at T=310 K.

Dendrimer	$R_{g}$ [nm]	$R_{h}$ [nm]	$R_{h}/R_{g}$	$\sqrt{5/3}R_{g}$ [nm]	$R_{e}$ [nm]	$R_{\max}$ [nm]	α
Lys-2Lys	2.04	1.68	0.83	2.63	2.71	3.14	0.02
Lys-2Gly	1.27	1.12	0.89	1.64	1.93	2.43	0.01

**Table 4:** The local characteristics of Lys-2Lys and Lys-2Gly dendrimers: the average number of inter-dendrimer ($\langle n_{H}^{id}\rangle$) and water–dendrimer ($\langle n_{H}^{dw}\rangle$) hydrogen bonds, the average number of ion pairs $\langle n_{ion\;pairs}\rangle$ between charged groups in dendrimer and counterions, the effective dendrimer charge Q*, the relative effective dendrimer charge $Q*/Q_{bare}$, the surface charge density σ, and ζ potential at T=310 K (the average body temperature).

Dendrimer	$\langle n_{H}^{\mathrm{id}}\rangle$	$\langle n_{H}^{\mathrm{dw}}\rangle$	$\langle n_{\mathrm{ion}\;\mathrm{pairs}}\rangle$	Q* (e)	$Q^{*}/Q_{\mathrm{bare}}$	σ (e/nm${}^{2}$)	ζ (mV)
Lys-2Lys	1.52	179.17	5.69	13.18	0.30	0.11	17.27
Lys-2Gly	7.68	100.68	0.67	9.39	0.59	0.13	20.03
